# Effect of Indigofera aspalathoides on the Expression of Inducible Nitric Oxide Synthase During 7,12-Dimethylbenz[a]anthracene-Induced Hamster Buccal Pouch Carcinogenesis

**DOI:** 10.7759/cureus.71841

**Published:** 2024-10-19

**Authors:** Philips Abraham, Sachu Philip, Salman Azad Mohammed, Hafsa Mohammed Unni

**Affiliations:** 1 Department of Biochemistry, Al Azhar Medical College Hospitals and Research Institute, Thodupuzha, IND; 2 Department of Biochemistry, Swamy Vivekanandha Medical College Hospitals and Research Institute, Namakkal, IND; 3 Department of General Medicine, Al Azhar Medical College Hospitals and Research Institute, Thodupuzha, IND

**Keywords:** carcinoma, dmba, indigofera aspalathoides, inos, keratinocytes, nitric oxide

## Abstract

Background: The mechanisms of inflammation and carcinogenesis are significantly influenced by nitric oxide (NO). Three different kinds of nitric oxide synthase (NOS) have been previously found: neuronal NOS, inducible nitric oxide synthase (iNOS), and endothelial NOS.

Objectives: This study looked into the properties of iNOS in hamster buccal pouch carcinogenesis caused by 7,12-dimethylbenz[a]anthracene (DMBA).

Methods: Thirty outbred young male Syrian golden hamsters, aged six weeks, were split into five groups at random: control (n=6), administered with ethanolic extract (n=6), DMBA+EIA (ethanolic Indigofera aspalathoides) (n=6), 0.5% solution of DMBA in liquid paraffin (n=6), and DMBA alone (n=6).

Result: In the group treated with DMBA, the mean quantity of iNOS positive foci per section was roughly 12.2+/-4.7. The DMBA-treated pouch keratinocytes showed both nuclear and cytoplasmic stainings. Neither the mineral oil-treated nor the untreated pouches (n=10) showed any signs of iNOS activity.

Conclusion: In conclusion, this work has shown that hamster pouch carcinomas induced by DMBA express iNOS. This finding raises the possibility that the emergence of chemically induced oral carcinomas is linked to iNOS expression.

## Introduction

A total of 80-90% of cancer cases are caused by environmental risk factors such as radiation, chemicals, and viruses. The majority of chemicals do not show their genotoxicity on their own; instead, they need to be metabolically activated by several enzymes involved in drug metabolism. A highly reactive ultimate carcinogen can be explained by deoxyribonucleic acid (DNA) damage that leads to altered gene expression and changes the sequence of DNA [1]. Angiogenesis, which is essentially a four-step process that involves endothelial cell proliferation, endothelial cell motility through the extracellular matrix leading to capillary differentiation, and angiogenic stimulation, plays a significant role in the advancement of cancer. Tissues of basement membranes are first locally damaged, resulting in rapid disintegration and hypoxia. Following that, angiogenic factors cause endothelial cells to move. The third stage involves the proliferation and stabilization of endothelial cells, followed by the continued influence of angiogenic agents on the angiogenic process [2]. Newly developed blood arteries aid in the cancer cells' metastatic spread.

Many growth factors that are produced by tumor cells in the local environment play a crucial role in angiogenesis, which is a crucial step in the growth and spread of tumors [3]. Solid tumors undergo a vascular or angiogenic phase after a relatively inactive prevascular phase, which provides a sufficient supply of nutrients for the fast proliferation of the malignant population. This is supported by both experimental and clinical evidence. Angiogenesis is controlled by substances that serve as both activators and inhibitors. It is also crucial to downregulate vascular growth inhibitors and upregulate the action of angiogenic factors [4].

Research has indicated that nitric oxide (NO) production and inducible nitric oxide synthase (iNOS) expression are two angiogenic variables that are significant predictive indicators in the evaluation of tumor progression. New vascular growth is mediated by iNOS via NO, which serves as a cellular signal for angiogenesis. Vascular endothelial growth factor (VEGF) is an essential component of lymph and pathologic angiogenesis. Vascular permeability factor or VEGF is a protein that can trigger a series of angiogenic processes [5].

The nuclear element NF-kB, also known as kappa B, is a significant inducible transcription factor that controls the transcription of genes that promote and develop carcinogenesis, inflammation, and cancer. The tumor suppressor gene p53 is essential for preventing DNA damage and other physiological stressors, mostly by causing cell cycle arrest or apoptosis. Thus, the p53 gene stands for the protector of the human genome [6].

Despite massive efforts to find a cure, cancer continues to be a major public health concern. Patients' post-treatment quality of life is reduced, chemoresistance is on the rise, and secondary cancers are recurring [7]. Chemoprevention is thought to be an alternative, more practical, and essential approach to managing this terrible illness to overcome it. In addition to antioxidant vitamins, a variety of nonnutritive compounds included in a plant-based diet that are collectively referred to as phytochemicals have been found to have chemopreventive properties. According to Wattenburg, the majority of chemopreventive drugs work by (1) preventing the carcinogen from being formed from its precursor, (2) improving the carcinogen's detoxification by altering cellular metabolism, or (3) preventing the ultimate electrophilic or carcinogenic species from interacting with crucial cellular target molecules [8].

Chemical agents such as polycyclic aromatic hydrocarbon (PAH) seem to be the primary causative factor of oral cancer, and animal tumor models created by carcinogens are necessary to evaluate the effectiveness of novel therapy strategies [9]. A hamster buccal pouch (HBP) combined with topical treatment of a chemical carcinogen (7,12-dimethylbenz[a]anthracene (DMBA), a member of the PAH family, is the best model for studying mouth cancer, due to its high reproducibility, ease of monitoring, and close resemblance to human oral squamous cell carcinoma (OSCC). The HBP model offers precise control over experimental conditions, facilitating the study of carcinogenesis and therapeutic interventions [10].

The inspiration for this study stems from the need to find effective chemopreventive agents that can target the angiogenic pathways critical for cancer progression. Our focus on Indigofera aspalathoides (IA) was driven by its historical use in traditional medicine and its potential anti-cancer properties suggested by preliminary studies [8,10]. We aimed to understand how IA modulates angiogenesis, particularly through the regulation of iNOS, a key player in the angiogenic process. By using the DMBA-induced HBP model, we sought to replicate oral carcinogenesis in a controlled environment, allowing us to observe the specific effects of IA on the angiogenesis pathway. This study not only contributes to the growing body of knowledge on chemoprevention but also explores the potential of IA as a natural therapeutic agent, which could lead to the development of new, less toxic cancer treatments. Overall, our approach aimed to examine how IA affects the angiogenesis process by examining iNOS expression during DMBA-induced buccal pouch carcinogenesis in hamsters.

## Materials and methods

For the study, DMBA was used as a chemical and reagent. It was purchased from Sigma Chemical Company, St. Louis, MD. All the other chemicals used were of analytical grade.

The leaves of IA were shade-dried and ground into powder after being obtained from Thirunelveli District in Tamil Nadu. Petroleum ether was used to treat the powder to dewax it and remove the chlorophyll. The extract was collected using 95% alcohol and packed in a Soxhlet device. It was then concentrated under a vacuum and dried in a desiccator. It was suspended in 2% tween 20 for our study.

They were kept in conformity with the National Institute of Nutrition's requirements at the Indian Council of Medical Research (ICMR), Hyderabad, India. They were given an ad libitum supply of water and a standard pellet diet, along with standard temperature and humidity levels and a 12-hour light-dark cycle. The Animal Ethical Committee at Annamalai University accepted the study design with approval number 474-160/1999/CPCSEA.

**Figure 1:**
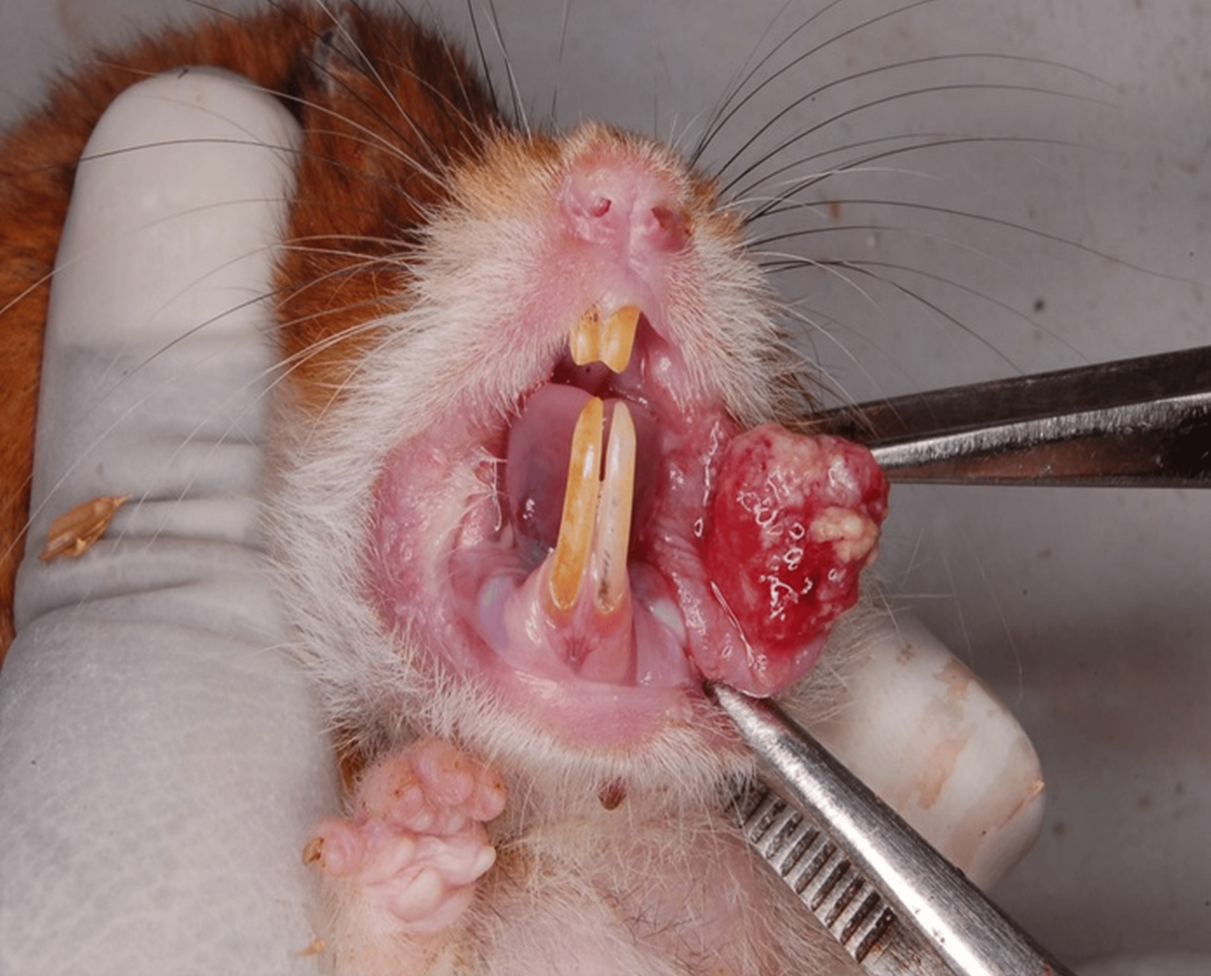
Syrian golden hamsters used in the study

A study was done on male Syrian Golden hamsters of 10-12 weeks weighing between 80 g and 100 g. 30 Hamsters were randomized into experimental and experimental design control groups and divided into five groups of six animals each (Figure 1; Table 1). The standard drug treatment group was not there in our study since there is no specific drug of choice for squamous cell carcinoma.

**Table 1:** Allocation of the groups DMBA: 7,12-dimethyl benz[a]anthracene; EIA: ethanolic Indigofera aspalathoides

Groups	Description
Group I	Control
Group II	Animals were administered with ethanolic extract alone
Group III	DMBA+EIA (an intra-gastric administration of 250 mg/kg body weight of ethanol extract from leaves of IA on the days alternate to DMBA)
Group IV	Painted three times per week with 0.5% solution of DMBA in liquid paraffin with a number 4 brush
Group V	DMBA alone

After an overnight fast, all selected animals were sacrificed via cervical dislocation when the experiment came to an end at 14 weeks. Using a modified Griess-Ilosvay reaction, the amount of NO radical was determined [11]. 

The mean ± standard deviation is used to express data. Analysis of variance was used to do statistical analysis on the results from biochemical assays, and the least significant difference test was used to compare group averages. At P<0.01, the results were deemed statistically significant.

## Results

Expression of protein markers was regarded as negative (0) when there was no staining; weak (1) when the staining was focal and mildly intense; moderate (2) when between one-third and two-thirds of cells stained moderately; and strong (3) when the majority of cells (>two third) stained intensely (Table 2).

**Table 2:** Distribution pattern on expression of Bax, Bcl-2, p53, iNOS, VEGF, and PDGF in control and experimental animals Bax: Bcl-2-associated X protein; Bcl-2: B-cell lymphoma 2; p53: tumor protein p53; iNOS: inducible nitric oxide synthase; VEGF: vascular endothelial growth factor; PDGF: platelet-derived growth factor

Group	Bax				Bcl-2				p53				iNOS				VEGF				PDGF			
	0	1	2	3	0	1	2	3	0	1	2	3	0	1	2	3	0	1	2	3	0	1	2	3
I	5	1	-	-	6	-	-	-	6	-	-	-	6	-	-	-	6	-	-	-	6	-	-	-
II	6	0	-	-	5	1	-	-	6	-	-	-	5	1	-	-	6	-	-	-	6	-	-	-
III	5	1	-	-	5	1	-	-	6	-	-	-	5	1	-	-	5	1	-	-	6	-	-	-
IV	3	3	-	-	2	4	-	-	6	-	-	-	3	3	-	-	3	3	-	-	2	4	-	-
V	3	3	-	-	1	5	-	-	6	-	-	-	2	4	-	-	2	4	-	-	4	2	-	-

**Figure 2:**
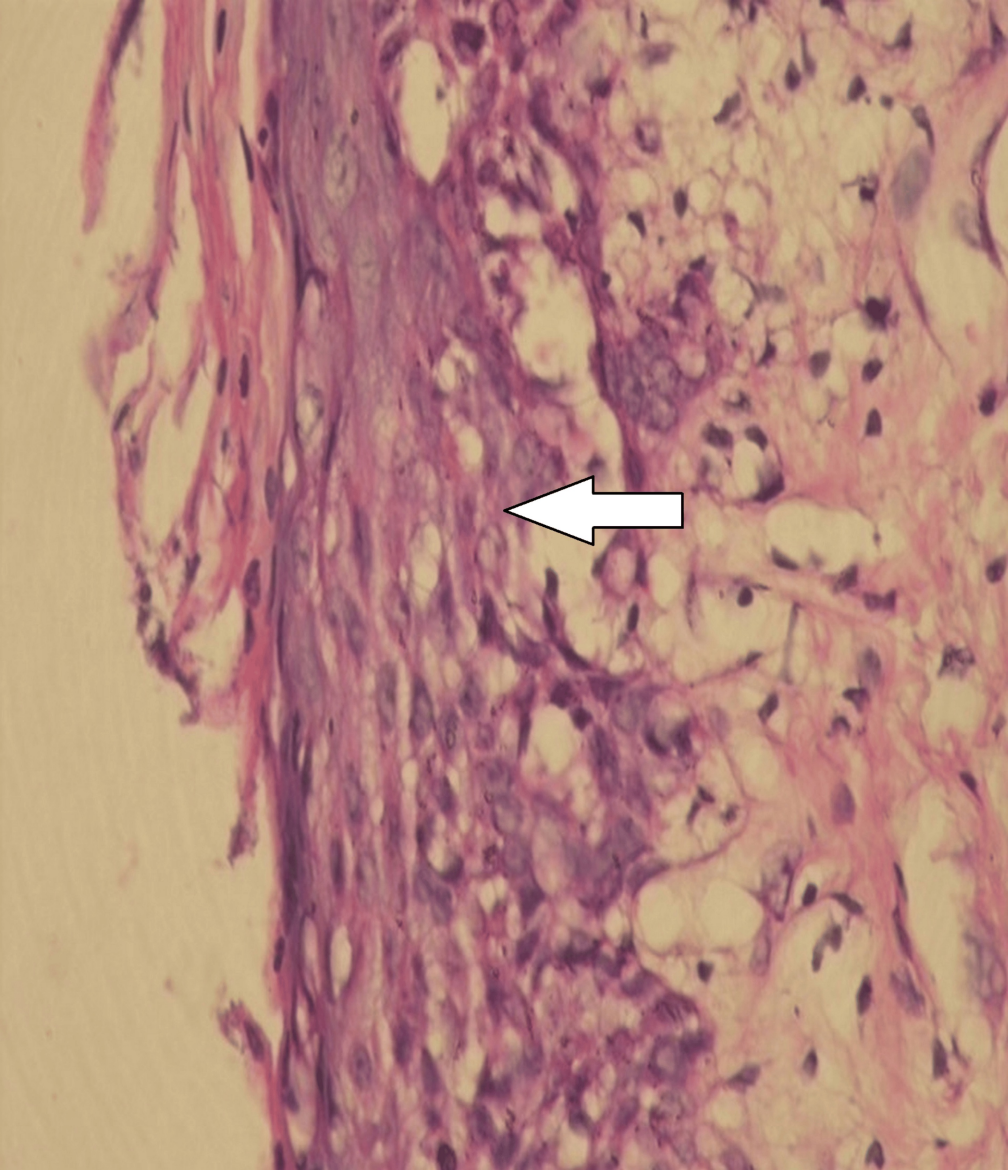
Immunoexpression pattern of p53 proteins observed buccal mucosa p53: tumor protein p53

**Figure 3:**
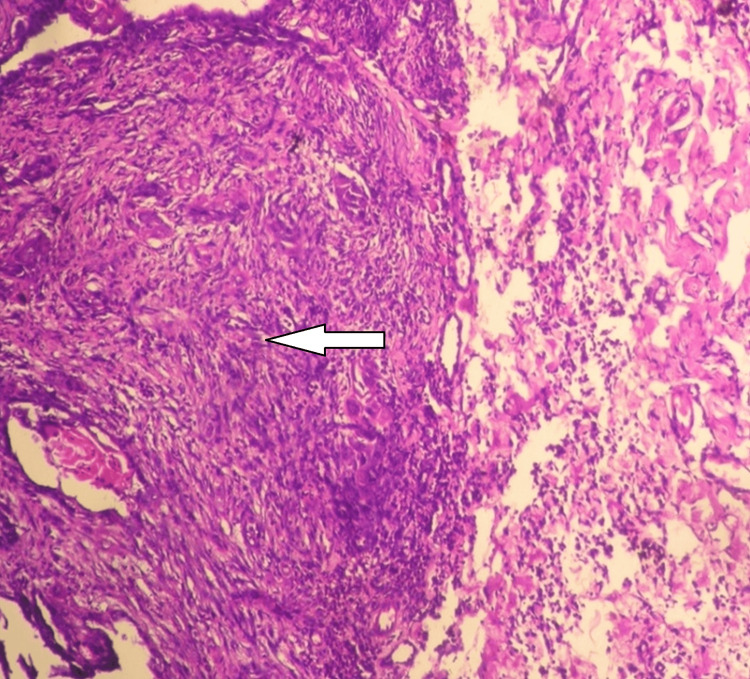
Immunoexpression pattern of pCNA proteins pCNA: proliferating cell nuclear antigen

**Figure 4:**
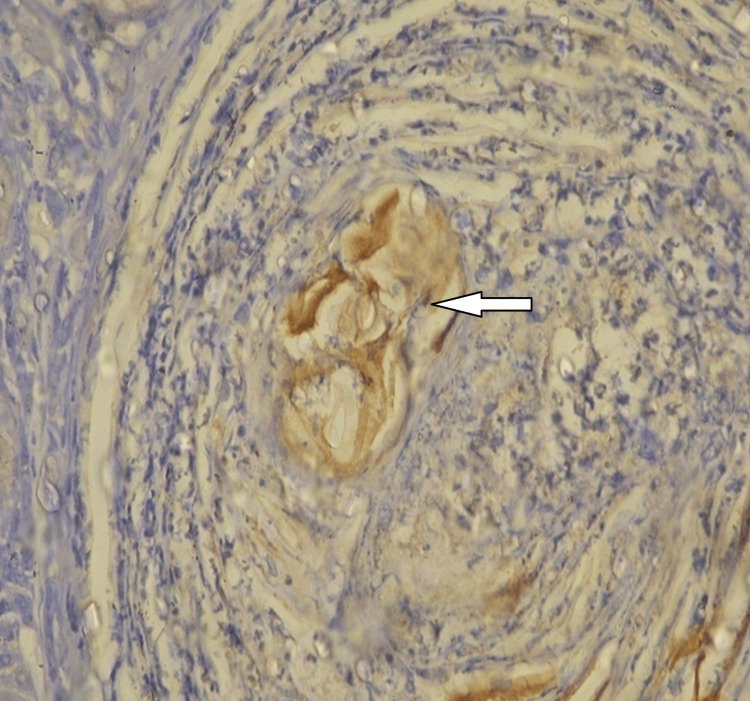
iNOS expression in experimental hamsters iNOS: inducible nitric oxide synthase

**Figure 5:**
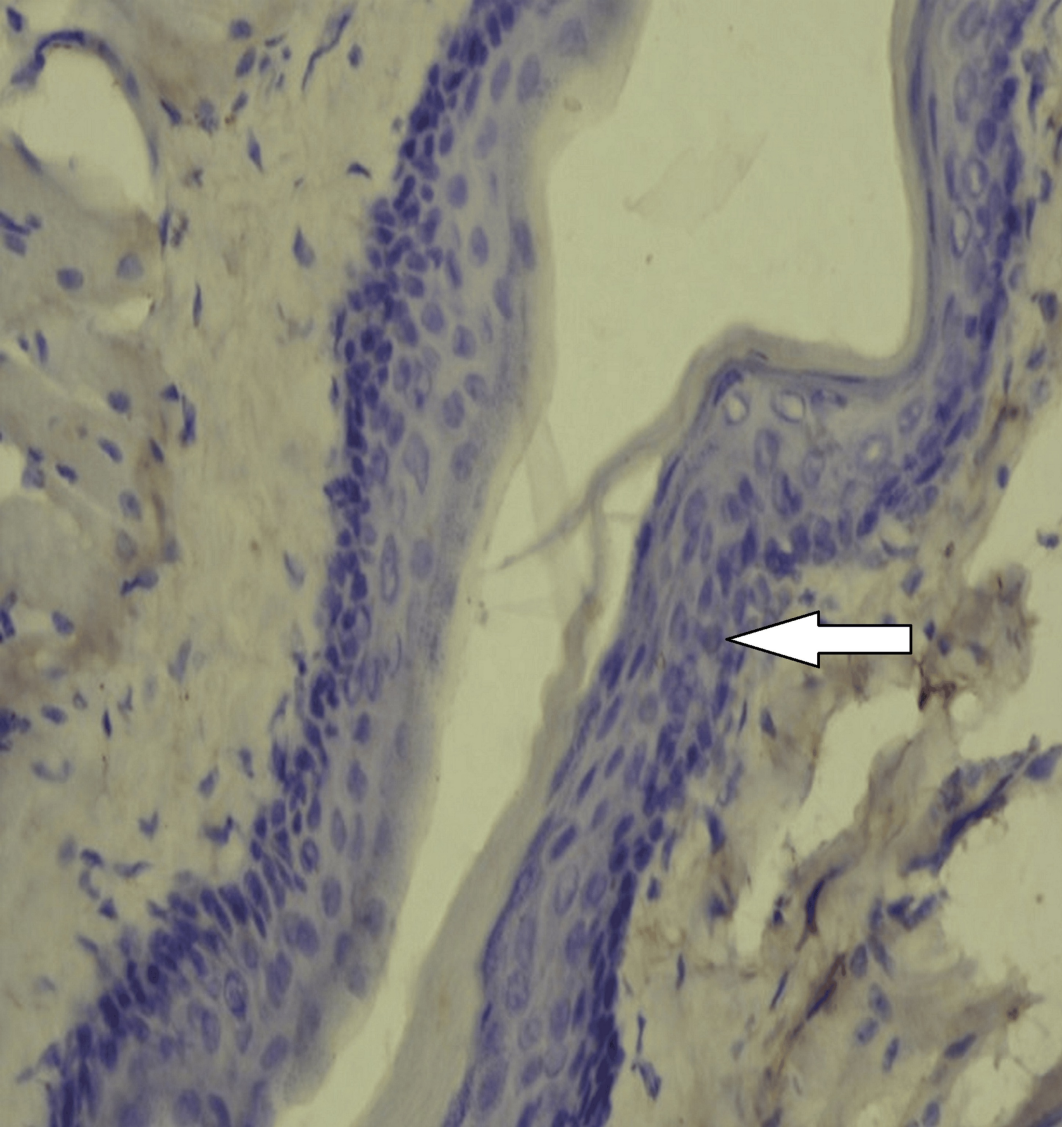
Immunoexpression pattern of VEGF proteins VEGF: vascular endothelial growth factor

The data indicates varying levels of protein expression in response to different treatments. Group I (Control) consistently shows no or very low expression across all proteins, establishing a baseline. Groups II and III, which involve the administration of ethanolic extract or combined treatments, show low expression levels for Bax, Bcl-2, iNOS, and VEGF, indicating a potential regulatory effect of the treatments on these proteins. Groups IV and V, involving DMBA exposure, show increased expression of angiogenesis-related proteins (VEGF and PDGF) and apoptosis-related proteins (Bax and Bcl-2), highlighting the impact of DMBA on these pathways and its role in carcinogenesis and angiogenesis. The presence of low expression in these groups suggests a heightened response to the carcinogenic stimulus. The immunoexpression pattern of p53 proteins (tumor protein p53), pCNA (proliferating cell nuclear antigen) proteins, iNOS, and VEGF proteins histopathologically is seen in Figures 2-5.

## Discussion

The multi-step process of angiogenesis, involving the proliferation, migration, and differentiation of endothelial cells, is regulated by an intricate balance between angiogenic inducers and inhibitors [1]. Tumor cells, for their survival, produce various physiologically active angiogenic agents. Consequently, the degree of angiogenesis and the expression of angiogenic factors can serve as biomarkers for tumor growth and metastasis [2]. Examining these variables offers new insights into the medicinal and chemopreventive potential of phytochemicals. iNOS is one such enzyme responsible for producing NO, a powerful modulator of vascular permeability. It has been demonstrated that iNOS expression and NO generation contribute to cancer growth and invasion through its angiogenic mechanisms. Immunohistochemistry (IHC) analysis revealed significantly higher levels of iNOS expression in the buccal pouch of mice treated with DMBA alone, consistent with previous findings [12]. The primary transcriptional regulator of the iNOS gene is NF-kB, and its transcriptional regulation occurs mainly at the transcriptional level. Therefore, the elevated activated NF-kB levels observed in the animals receiving DMBA alone may account for the high iNOS level. Additionally, p53 has been observed to inhibit iNOS expression; however, this inhibitory effect may not have been effective due to altered p53, leading to increased iNOS [13]. Conversely, DMBA, ethanolic Indigofera aspalathoides (EIA), and CIA administrations reduced iNOS expression. Certain chemopreventive phytochemicals control the overexpression of iNOS by inhibiting NF-kB [5,6]. EIA and CIA in our investigation also prevented NF-kB activation, potentially leading to the downregulation of iNOS expression.

Platelet-derived growth factor (PDGF), a powerful mitogen and chemotactic factor for various cells, promotes tumor cell growth autocrine and is implicated in cancer progression [14]. PDGF signaling regulates tumor growth autocrine and affects stroma cells paracrine, as seen in small cell, melanoma, and breast cancers. Elevated PDGF levels have been found in several malignancies, and its level is positively correlated with blood vessel density during neovascularization. Ets-1, also known as the Winged Helix-Turn-Helix Factor, controls PDGF [15]. Reactive oxygen species (ROS) increase Ets-1 expression, located in the PDGF promoter. The elevated PDGF expression seen in the buccal pouch of DMBA-treated animals may result from significant oxidative damage caused by carcinogen exposure. Elevated PDGF levels stimulate angiogenesis by increasing VEGF, MMP, and E-cadherin expression. Natural chemopreventive substances, such as indole compounds, have been proposed to prevent tumor growth and angiogenesis by inactivating the PDGF pathway. IHC examination revealed mild to negative PDGF expression in the buccal pouch of rats that received DMBA and EIA or CIA [16]. Antioxidant phytochemicals in the extract may affect ROS-mediated transcription process activation, accounting for this remarkably low PDGF level.

VEGF stimulates angiogenesis in vitro and in vivo and is a mitogen specific to endothelial cells. VEGF, also known as vascular permeability factor, induces cascades of angiogenic processes and may serve as a marker for tumor invasion and metastasis in various malignancies. In this study, tumor-bearing mice had noticeably higher VEGF expression levels compared to other groups. VEGF expression has been shown to be upregulated by hypoxia-inducing factor (HIF), NO, PDGF, and mutant p53, contributing to the increased VEGF levels. CIA- or EIA-treated animals had low VEGF expression levels in their buccal pouches, according to IHC analysis (Groups IV and V). Chemopreventive substances like green tea polyphenols, red wine, curcumin, resveratrol, and neem fractions inhibit VEGF expression. Previous research suggests these phytochemicals exhibit this characteristic by lowering iNOS expression or preserving p53's functional state (preventing mutation). Certain drugs influence the HIF pathway to inhibit VEGF expression. Our investigation revealed that the CIA and EIA considerably reduced iNOS expression while preserving the functional state of p53. Therefore, their effect on iNOS, PDGF, and p53 expression may account for the observed downregulation of VEGF expression by EIA and CIA.

The group of rats induced with DMBA alone had considerably higher levels of the angiogenic factors of VEGF, PDGF, and iNOS. The mice co-treated with CIA or EIA showed a significant decrease in the production of the aforementioned proteins, indicating the anti-angiogenic properties of the IA extracts.

The study's limitations include a relatively small sample size of 30 outbred young male Syrian golden hamsters, potentially limiting the generalizability of findings. While Syrian golden hamsters are commonly used in carcinogenesis research, extrapolating results to humans should be done cautiously. The study's focus on iNOS expression in HBP carcinogenesis induced by DMBA may not fully represent the multifactorial nature of carcinogenesis, excluding factors like genetic predisposition and immune response. Additionally, the short-term observation period might not capture the long-term effects of iNOS expression on carcinogenesis. Furthermore, the study's findings are specific to the experimental conditions and animal model, necessitating cautious extrapolation to other settings. Addressing these limitations through larger sample sizes, diverse animal models, longer observation periods, and comprehensive investigation of additional factors could enhance the understanding of iNOS's role in carcinogenesis and inflammation.

## Conclusions

The group of rats induced with DMBA alone had considerably higher levels of the angiogenic factors of VEGF, PDGF, and iNOS. The mice co-treated with chemiluminescence immunoassay and enzyme immunoassay showed a significant decrease in the production of the aforementioned proteins, indicating the anti-angiogenic properties of the IA extracts. These findings demonstrated the lethal ability of IA extracts, which may be explained by the cancer cells' pro-oxidant characteristics. The current study's observation clearly shows that the CIA and EIA may have anticancer effects.
